# Effect of central and peripheral cone- and rod-specific stimulation on the pupillary light reflex

**DOI:** 10.1007/s10792-021-02132-1

**Published:** 2021-11-26

**Authors:** Anton Sonntag, Carina Kelbsch, Ronja Jung, Helmut Wilhelm, Torsten Strasser, Tobias Peters, Krunoslav Stingl, Barbara Wilhelm

**Affiliations:** 1 Pupil Research Group, Centre for Ophthalmology, University of Tübingen, Elfriede-Aulhorn-Str. 7, 72076 Tübingen, Germany; 2 University Eye Hospital, Centre for Ophthalmology, University of Tübingen, Tübingen, Germany; 3 Institute for Ophthalmic Research, Centre for Ophthalmology, University of Tübingen, Tübingen, Germany; 4 Centre for Rare Eye Diseases, University of Tübingen, Tübingen, Germany

**Keywords:** Pupil campimetry, Pupillography, Cones, Rods, Centre, Periphery

## Abstract

**Purpose:**

To assess the effect of central and peripheral stimulation on the pupillary light reflex. The aim was to detect possible differences between cone- and rod-driven reactions.

**Methods:**

Relative maximal pupil constriction amplitude (relMCA) and latency to constriction onset (latency) to cone- and rod-specific stimuli of 30 healthy participants (24 ± 5 years (standard deviation)) were measured using chromatic pupil campimetry. Cone- and rod-specific stimuli had different intensities and wavelengths according to the Standards in Pupillography. Five filled circles with radii of 3°, 5°, 10°, 20° and 40° and four rings with a constant outer radius of 40° and inner radii of 3°, 5°, 10° and 20° were used as stimuli.

**Results:**

For cone-and rod-specific stimuli, relMCA increased with the stimulus area for both, circles and rings. However, increasing the area of a cone-specific ring by minimizing its inner radius with constant outer radius increased relMCA significantly stronger than the same did for a rod-specific ring. For cones and rods, a circle stimulus with a radius of 40° created a lower relMCA than the summation of the relMCAs to the corresponding ring and circle stimuli which combined create a 40° circle-stimulus. Latency was longer for rods than for cones. It decreased with increasing stimulus area for circle stimuli while it stayed nearly constant with increasing ring stimulus area for cone- and rod-specific stimuli.

**Conclusion:**

The effect of central stimulation on relMCA is more dominant for cone-specific stimuli than for rod-specific stimuli while latency dynamics are similar for both conditions.

**Supplementary Information:**

The online version contains supplementary material available at 10.1007/s10792-021-02132-1.

## Introduction

Pupil campimetry, full-field pupillography and multifocal pupil perimetry are increasingly being employed in the clinic and are under further investigation by several research groups as methods to detect retinal and optic neuropathies such as glaucoma, age-related macular degeneration, retinitis pigmentosa and diabetic retinopathy in type 2 diabetes [1–11].

The use of specific wavelengths, stimulus durations and adaptation states at different locations of the visual field allows responses from rods, cones and intrinsically photosensitive retinal ganglion cells to be studied by pupil campimetry [12]. Based on several studies using chromatic pupillography that detected changes of the pupillary light reflex (PLR) to explicitly cell-addressing stimuli in patients with retinal diseases [13, 14] and glaucoma [15] the Pupil Research Group at the Centre for Ophthalmology developed a new type of device, the Chromatic Pupil Campimeter (CPC): a combination of cell-specific stimuli and a new pupillographic campimetry device introduced by Stingl et al. [1]. Kelbsch et al. presented this objective method to measure pupil responses separately for rods and L-cones at different locations in the visual field [2] and found that the mean relative maximal constriction amplitude (relMCA) caused by cone-specific stimuli was larger in the centre of the retina and decreased in a hill-shaped form towards the periphery. Rod-specific relMCAs were smaller and showed a flatter profile around the retina with only a minor peak in the centre. Patients with rod or cone deficiencies showed no rod- and severely impaired cone-specific pupillary reactions respectively [2].

Despite the arising use of pupillography still only little is known about the characteristics of pupillomotor receptive fields, especially those of photoreceptor-specific stimulation.

Skorkovska et al. were the first to explicitly examine summation effects within the pupillary pathway using white light and found that pupillomotor receptive fields are larger than receptive fields for visual perception, that their size increases with increasing retinal eccentricity and that their size decreases with increasing brightness [16].

It is well-known that the amplitude of the PLR is linearly related to the logarithm of the stimulus intensity [17–19]. The light-adapted pupil size depends on the product of stimulus luminance and area (corneal flux density, CFD) [20, 21]. Hu et al. found a correlation between the maximum constriction amplitude, eccentricity and CFD when using white peripheral stimuli [22]. In contrast, Park et al. investigated the relationship between the CFD and the PLR using central stimuli and found that only rod- and melanopsin-mediated pupillary responses were CFD-dependent. On the other hand, they found that cone-mediated responses depended only on stimulus luminance and not on stimulus size [23].

Previous studies have shown an eccentricity effect on the PLR to local stimuli with a decrease from the centre to the periphery for white light [1, 16, 19, 22, 24] and also for dim blue and red light under rod- and cone-specific conditions [2, 25]. The eccentricity effect in these studies was larger for cone-specific stimulation than for rod-specific stimulation. However, Joyce et al. did not observe a difference in the relMCA for corneal flux density equated photopic short- and long-wavelength stimuli presented at 0° and 20° eccentricity [26].

Regarding this limited state of scientific knowledge about summation effects and the observed eccentricity effect on the PLR, we wanted to gain more insight into this topic, particularly on the effect of central versus peripheral stimulation, which is relevant for clinical use of pupillography. Our study aimed to assess the effect of central and peripheral cone- and rod-specific stimulation on the pupillary light reflex in a clinically applicable set-up and to detect differences between cone and rod influence using CPC [1, 2].

## Methods

### Participants and ethical aspects

The inclusion criteria were: healthy participants between 18 and 50 years, no ocular disease, no medication that could affect the pupillary light reflex and a refraction error less than or equal to ± 3 D. To exclude any pathological findings, each participant underwent a medical anamnesis and an ophthalmological examination at the neuro-ophthalmology unit of the University Eye Hospital Tübingen, including visual acuity test, swinging-flashlight-test, slit-lamp-examination and funduscopy, optical coherence tomography (OCT) (Spectralis-OCT; Heidelberg Engineering GmbH, Germany) and 30° visual field examination (static automated strategy, Octopus 101 or 900; Haag-Streit International, Wedel, Germany). Thirty healthy volunteers (14 males, 16 females) with a mean age of 24 ± 5 years (standard deviation) were enrolled in the study. They were recruited either from the staff of the University Eye Hospital Tübingen or from the student body of the University of Tübingen. All participants received detailed information about the study and its aims and gave their written informed consent. The study was approved by the local institutional ethics committee (project-number: 775/2016BO2) and obeyed the tenets of the Declaration of Helsinki.

### Chromatic pupil campimetry set-up

**Fig. 1:**
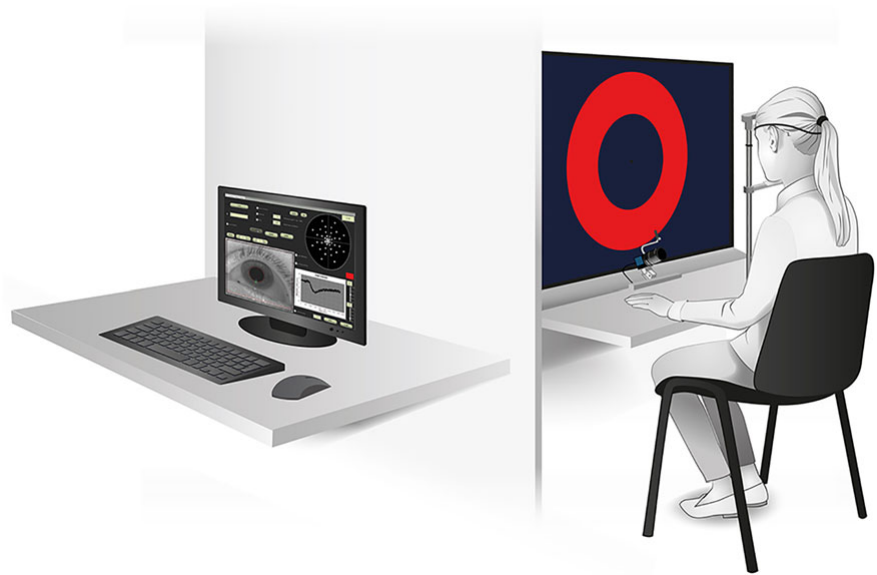
The setup of the examinations: participants sat on a comfortable chair and stimuli were presented on a large monitor while the examiner sat in front of a darkened computer monitor behind a partition wall

A modified version of the CPC as described by Kelbsch et al. [2] was used to specifically stimulate predominantly rods or cones, respectively. The experiment was performed in a completely dark and quiet room of the University Eye Hospital and was about one hour in duration. The participants sat on a comfortable chair in front of an OLED-monitor (LG OLED 55C7V), located at a distance of 40 cm in front of the subject’s eye and presenting the stimuli (see Fig. 1). The head was placed in a combined chin- and headrest for stabilization and comfort. Only the left eye was examined while the right eye was covered by an eyepatch. An infrared camera (DMK23UV024, The Imaging Source GmbH) with a temporal resolution of 10 ms and a 50-mm TV-lens 1:1.4, located below the screen, recorded the pupil continuously. The pupil diameter was calculated online by a Java™-based in-house software described by Stingl et al. [1] using a modified Starburst algorithm [27] that detected the black pupil’s edge from the image captured by the camera. An ellipse was fitted to the points of the detected edge using a random sample consensus (RANSAC) approach [28] and the pupil centre and diameter were determined in real-time [1]. The estimated error of the algorithm was approximately 1–2 pixels which correspond to the estimated error of 0.1 mm per image. A dim fixation point (0.01 cd/m^2^) with a radius of 1° was presented in the centre of the OLED screen.

### Stimulus characteristics

Stimuli were created by an in-house developed software as described by Stingl et al. [1]. We used cone- and rod-specific stimuli whose intensity was modified according to Kelbsch et al. [2]. The intention was not to drive the M. sphincter pupillae to its mechanical limits despite a relatively large stimulus size. L-cone-specific stimuli (photCPC) had a luminance of 4 cd/m^2^ and a wavelength of 620 ± 30 nm full width at half maximum (FWHM). Stimulus duration was 1000 ms and stimuli were presented on a dim blue background with a luminance of 0.01 cd/m^2^ and a wavelength of 460 ± 30 nm FWHM. The test was applied after 10 min of light adaptation to the background to partially saturate the rods [29]. Rod-specific stimuli (scotCPC) had a luminance of 0.001 cd/m^2^ and a wavelength of 460 ± 30 nm FWHM. Their stimulus duration was 100 ms and they were presented on a completely dark background (0 cd/m^2^) after 20 min of dark adaptation.

The energy and spectrum of our stimuli were measured at the position of the proband’s eye using an LED-Spectrometer (MK-350S-Premium, UPRTek, Taiwan). The results are shown in supplementary Fig. S1. Luminance, spectrum and duration of our stimuli are in accordance with the ISCEV Standard for (cone- and rod-specific) full-field clinical electroretinography [30] as well as the Standards in Pupillography [29]. The background during and before the application of cone-specific stimuli was used to partially saturate rods.

The baseline period before the first stimulus presentation for all stimuli was 500 ms and the interstimulus interval was 4500 ms. The baseline pupil size was calculated for each step individually. A stimulus was repeated automatically if a blink occurred during the presentation or if at least 90% of the initial pupil diameter was not reached before the following stimulus presentation.

**Fig. 2:**
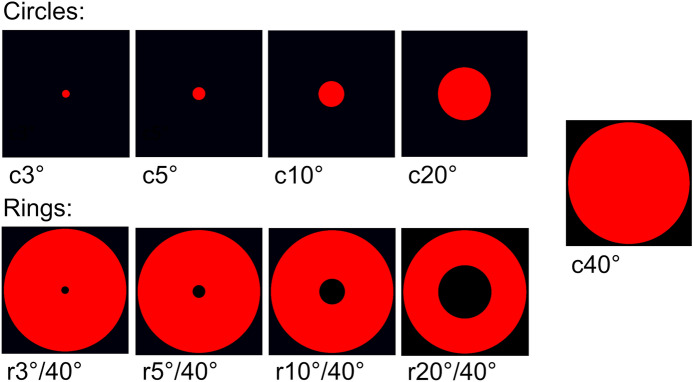
Shapes of applied stimuli. This figure shows the red versions (cone-specific stimuli, photCPC; scotopic stimuli used blue colour). There were 9 different stimuli for either cones or rods and 10 repetitions. Below the stimuli, the abbreviations are given which are used hereinafter

There were 9 different stimuli for either rods or cones and each one was repeated 10 times. Stimuli consisted of five filled circles with a radius of 3°, 5°, 10°, 20° and 40° and four rings with a constant outer radius of 40° and inner radii of 3°, 5°, 10° and 20° (see Fig. 2).

In each protocol the circles were presented first, beginning with 10 repetitions of the smallest circle and ending with 10 repetitions of the largest circle. Subsequently, the rings were presented, starting with the ring with the largest inner radius (smallest stimulated area) and ending with the ring with the smallest inner radius (largest stimulated area).

Table 1 shows the area of the different stimuli in deg^2^, calculated using the radii in degree and the formula *A* = *πr*^2^.

**Table 1:** The area of each stimulus in degrees visual angle^2^ (rounded to whole numbers) in phot CPC and scotCPC

Stimulus	Area (deg^2^)
c3°	28
c5°	79
c10°	314
c20°	1257
c40°	5027
r20°/40°	3770
r10°/40°	4712
r5°/40°	4948
r3°/40°	4998

### Data management and statistical analysis

Mean relative maximal pupillary constriction amplitude (relMCA) and mean latency to constriction onset (latency) to the different stimuli were determined (for more details see supplementary information, Fig. S2). For the further statistical analysis, one participant was excluded because his relative pupillary responses to the rod-specific stimuli lay two standard deviations below those of all the other participants. Data were visually inspected for following a normal distribution. Analyses of variance (ANOVA) for repeated measurements and Bonferroni corrected post-hoc tests (two-tailed paired t-test) were carried out between relMCAs as well as between latencies to the cone and rod-specific circle- and ring-stimuli. Additionally, a two-tailed paired t-test between the rod-and cone-specific difference of relMCA between the 20°/40° and the 3°/40° ring as well as one between the rod- and cone-specific latencies to the stimuli were performed. Furthermore, relMCAs to the corresponding ring and circle stimuli which combined create a 40° circle-stimulus were summed. An ANOVA for repeated measurements and a Bonferroni corrected post-hoc test (two-tailed paired t-test) were subsequently carried out between the different combinations of summation.

RelMCAs and latencies of 29 participants were analysed. *p*-Values smaller than 0.05 were considered statistically significant. Results are presented with mean ± standard deviation (SD).

## Results

### Baseline pupil diameter

The average baseline pupil diameter of the 29 participants during photCPC was 6.3 ± 1.1 mm and the average baseline pupil diameter during scotCPC was 7.4 ± 0.8 mm.

### Effect of increasing the stimulus: non-linear increase of pupil response with increasing stimulus area

#### Cone-specific protocol: photopic CPC (photCPC)

**Fig. 3:**
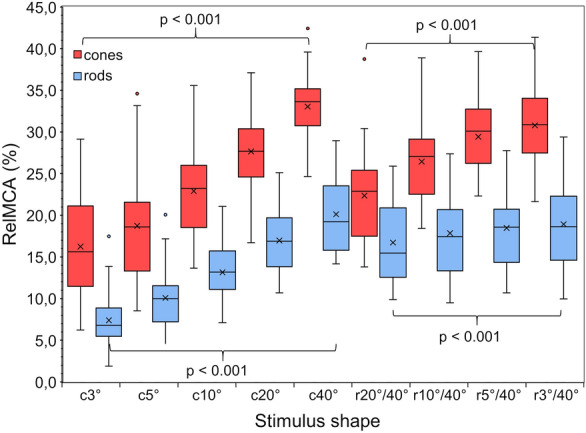
The average (of 10 pupil responses per participant) relMCAs (relative maximal pupillary constriction amplitudes) to the different stimuli are shown by the box-whisker plots (*n* = 29). The mean values are shown by the crosses. Responses to cone-specific stimuli are coloured red, responses to rod-specific stimuli are coloured blue. The most important significant differences mentioned in the text are shown by the braces. Shapes are explained in Fig. 2

In Fig. 3 the results of the cone and rod response to circle and ring stimuli are shown. For cones, the relMCA significantly increased with increasing the radius of the circle stimulus (*p* < 0.001). The response to a 3° circle was 16.2 ± 6.3% while the response to a 40° circle was 33.0 ± 4.1%. Likewise, the relMCA significantly increased with increasing area of the ring stimulus (*p* < 0.001): The response to a 20°/40° ring was 22.4 ± 5.9%, while the response to a 3°/40° ring was 30.8 ± 4.6%. The response to the increase of the stimulated area, whether circles or rings, was non-linear.

#### Rod-specific protocol: scotopic CPC (scotCPC)

The results for the rod-specific protocols (Fig. 3) showed overall smaller relMCAs than those for the photCPC.

As in the photCPC, the relMCA significantly increased with increasing radius of the circle stimulus and there was a significant effect of stimulus size on relMCA (*p* < 0.001). The response to a 3° circle was 7.4 ± 3.3%, while the response to a 40° circle was 20.1 ± 4.6%. Also, the relMCA significantly increased with increasing area of the ring stimulus, although the difference is only small: The response to a 20°/40° ring was 16.7 ± 5.0% while the response to a 3°/40° ring was 18.9 ± 5.1% (for both: *p* < 0.001). Nevertheless, the increase of the relMCA to rod-specific rings was statistically significant smaller than to cone-specific rings. There was an absolute difference of only 2.2 ± 1.7% between the 20°/40° and the 3°/40° ring for the rod-specific stimuli. For the cone-specific stimuli, the corresponding difference was significantly larger at 8.4 ± 3.4% (*p* < 0.001). The difference between the rod-specific relMCAs to the 10°/40° and the 5°/40° ring, as well as the difference between the ones to the 5°/40° and the 3°/40° ring, were small and non-significant.

The increase of the response to the rod-specific circles and rings was also non-linear.

Supplementary Fig. S3 shows some exemplary pupil traces of proband number 1.

For both, cones and rods, a circle stimulus with a radius of 40° created a significantly smaller relMCA (*p* < 0.001) than the summed response to the ring and circle stimuli which create a 40° circle stimulus if combined (e. g. c3° + r3°/40°) (see supplementary Fig. S4).

### Latency to constriction onset

**Fig. 4:**
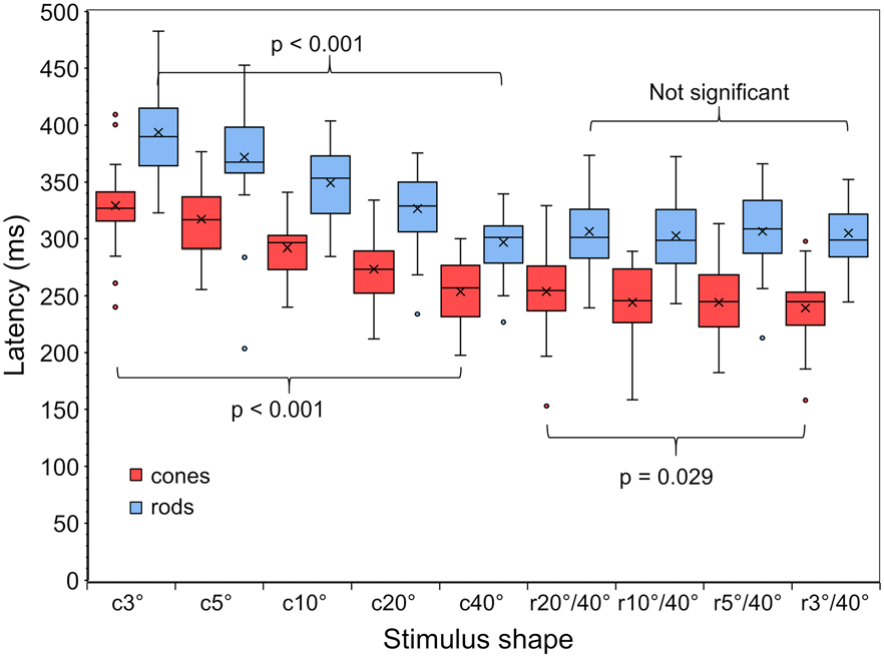
The average (of 10 pupil responses per subject) latencies to constriction onset to the circle and ring stimuli are shown by the box-whisker plots (n = 29). The mean values are shown by the crosses. Latencies to cone-specific stimuli are coloured red, latencies to rod-specific stimuli are coloured blue. The most important significant differences mentioned in the text are shown by the braces

In Fig. 4 we plot the latency to constriction onset for the cone and rod stimuli for the circle and ring stimuli.

#### PhotCPC: influence of stimulus configuration on latency

For the cone-specific stimuli (red boxplots in Fig. 4), the mean latency decreased significantly with increasing radius of circle stimulus. The effect of stimulus size on latency was significant (*p* < 0.001 for circles, *p* = 0.004 for rings). The latency to a 3° circle was 329 ± 34 ms while the latency to a 40° circle was 254 ± 28 ms (*p* < 0.001). In contrast, latency stayed nearly constant with increasing area of the ring stimulus, though the effect of a smaller inner radius was still significant. The latency to a 20°/40° ring was 254 ± 35 ms and different from the latency to a 3°/40° ring that was 239 ± 31 ms (*p* = 0.029).

#### ScotCPC: longer latencies

The results for the rod-specific stimuli (blue boxplots in Fig. 4) showed overall longer mean latencies to constriction onset than those to the photCPC (*p* < 0.001). The mean latency difference between rods and cones was 57 ± 32 ms. As in the photCPC, latency decreased significantly with a larger radius of circle stimuli. The effect of circle stimulus size on latency was significant (*p* < 0.001). The latency to a 3° circle was 394 ± 40 ms while the latency to a 40° circle was 297 ± 26 ms (*p* < 0.001).

In contrast, there was no significant decrease of latency with a larger area of the ring stimulus. The effect of ring stimulus size on latency is not significant. The latency to a 20°/40° ring was 306 ± 33 ms and the latency to a 3°/40° ring was 305 ± 31 ms (non-significant).

## Discussion

Our study focuses on the effect of central and peripheral stimulation on the pupillary light reflex. The aim was to detect possible differences between cone- and rod-driven reactions.

For both, cone- and rod-specific stimulation, relMCA increased with increasing stimulus size (see Fig. 3). This was expected as a larger stimulus activates a larger area of the retina. It is well-known that the amplitude of the PLR is linearly related to the logarithm of the stimulus intensity [17–19] but differing results on the relationship between PLR and stimulus size have been reported in the literature. Various studies have shown that the light-adapted pupil size depends on the product of luminance and stimulus area, the corneal flux density (CFD) [20, 21]. Hu et al. expressed the maximum pupil constriction to white peripheral stimuli as a function of CFD and eccentricity [22]. Joyce et al. examined the relMCA caused by long- and short-wavelength stimuli under conditions constant for either CFD, irradiance or stimulus-size [26]: their study revealed that for both wavelengths a constant CFD resulted in a relatively constant pupillary constriction. A CFD-dependency of the maximum pupil constriction agrees with our results as the relMCA of cone- and rod-mediated responses increased with increasing stimulus size and consequently a larger CFD.

Cone-specific stimulation revealed that the effect of central stimulation is dominant for the PLR: although the stimulated area is much larger, the relMCA to a 40° circle stimulus was only two times stronger than the relMCA to a 3° circle (see Fig. 3). Moreover, increasing the area of central stimulation while the periphery is also stimulated had a significant effect on the pupillary response as well: Minimizing the inner radius of a ring-stimulus increased the pupillary response amplitude by more than one third.

With rod-specific stimuli, the effect of central stimulation is less dominant for the PLR than with cone-specific stimuli. The relMCA to a 40° circle stimulus was three times as large as the relMCA to a 3° circle (versus two times as large in photCPC) (see Fig. 3). Furthermore, in contrast to the photCPC, the increase of amplitude to the increasing size of ring stimuli (smaller inner radius) was significantly smaller than for cone-specific stimuli. Increasing the area of central stimulation while the periphery was already stimulated increased the pupillary response amplitude only minimally. One could argue that this is not surprising because there are almost no rods in the centre of the retina [31], but according to Curcio et al., the size of the average diameter of the rod-free zone in the middle of the retina is only 1.25° [31] which is also the reason for a response to our c3°-rod-stimulus.

For cones and rods, a circle stimulus with a radius of 40° created a lower relMCA than the summation of the relMCAs to the corresponding ring and circle stimuli which combined create a 40° circle-stimulus. This indicates a non-linear summation.

In our recent publication on CPC [2] and a study by Haj Yahia et al. [25] a prominent role of cones in the centre of the retina has also been described, i.e. larger eccentricity effect for cones than for rods. In patients with AMD the relMCAs to local stimuli were lower than those of an age-matched healthy control group, especially for the centre of the visual field. In addition, the profile of responses over the retina was flatter [6]. Our results are in line with these studies, although their results were based on local stimulation. Skorkovska et al. examined summation effects of the pupillary light reflex to white light under conditions of light adaptation and found the amplitude of the PLR being related to size, intensity and retinal location. They observed an eccentricity effect with lower relMCAs in the periphery [16]. However, their results cannot simply be transferred to our photoreceptor-specific results as they used other stimulus shapes, white light and no photoreceptor-specific stimulation. Several other studies have found an effect of eccentricity on the PLR to white light [1, 19, 22, 24].

Regarding latency to constriction onset, it can be seen that latency was significantly shorter for cones than for rods (see Fig. 4).

The mean difference in latency between rods and cones was 57 ± 32 ms. This confirmed previously published results of our group [2] and those of other groups [32–34] reporting latency differences in the range of 20 ms—100 ms. In our previous paper (applying small focal stimuli) we found a cone-specific latency of 277 ± 25 ms and a rod-specific latency of 372 ± 13 ms [2] which is in accordance with the current results.

Latency differences between photCPC and scotCPC are most probably caused by differences in the retinal processing of the cone and rod system. According to current knowledge, a rod on-stimulation is either transferred from rod bipolar cells via AII amacrine cells to cone bipolar cells or directly via gap junctions from the rod to a cone and thereafter to a cone bipolar cell [35–39]. Consequently, the rod ON-pathway includes at least one additional cell compared to the cone ON-pathway.

Latency decreased with increasing radius of circle stimuli for cones and rods (see Fig. 4). The latency difference between the different steps of circle sizes in our study was very similar, no matter how large the change of area was. This indicates a non-linear relation to stimulus size while the relation to the logarithm of stimulus size is negatively linear. These observations are in line with the results of Cibis et al. and Hu et al.: Cibis et al. found shorter latencies for larger stimuli and additionally a negative linear relation between the logarithm of the stimulus intensity and the pupillomotor latency [40] and Hu et al. determined latency to be a function of CFD [22]. In ERG, a faster b-wave can be determined with increasing light for both rods and cones [34] which is consistent with our results as well. In contrast, latency remained nearly constant with increasing ring stimulus size for both photoreceptors. This effect indicates that peripheral retinal stimulation might fasten pupillary dynamics leading to a decreased latency in comparison to central stimulation. On the other hand, saturation due to stimulus size can be considered.

In conclusion, the main outcome of this study is that the effect of central stimulation on the relMCA is more dominant for cone-specific stimuli than for rod-specific stimuli while latency dynamics are similar for rods and cones despite their absolute latency difference.

The clinical relevance of our study is that smaller stimulus sizes in the range of 3°—5° as used in the CPC or multifocal approaches are necessary to detect central defects under rod-specific test conditions.

We are aware of certain limitations of our study. The pupillary light reaction is a complex process that receives its input not only from rods and cones but also from intrinsically photosensitive retinal ganglion cells (ipRGCs) and will therefore be influenced by diffuse bipolar cells and dopaminergic amacrine cells [29]. Nevertheless, our stimuli were designed to stimulate the photoreceptors as specifically as possible while keeping clinical practicability [2] and being in terms with the ISCEV- and Pupillography-Standards [29, 30]. Finally, we did predominantly address rods or cones by using different light levels, wavelengths and states of adaptation, but cannot claim an absolute separation of their inputs.

Furthermore, we cannot completely exclude the effect of light scatter. If there was an effect of scatter, it would be expected to be stronger for scotCPC than for photCPC, as rods are more sensitive than cones [41] though a lower stimulus intensity leads to less light scatter. However, as the average retinal diameter of the rod-free zone is only 1.25° and as there are even more rods than cones at an eccentricity of 3° according to Curcio et al. [31], it is unlikely that our strong response to the 3° circle is an effect of scattering. Additionally, none of our probands reported any blurred stimulus margin.

Another point is the effect of refractive errors. Minor refractive errors do not influence pupillary light responses in CPC. However, it is difficult to define a numerical limit. According to our experience from former studies, we believe the limit of ± 3 D is reasonable. Because we examine without corrective glasses, higher hyperopia might induce a pupillary near response. In younger subjects, an accommodative near response up to 3 D is very small, in most cases absent [42].

Finally, we evaluated the results of 29 healthy young participants. Whether the same effects apply to other age groups remains open.

## Code availability:

The in-house developed software that was used for pupil measurements is described by Stingl et al. [1] and its code is stored at the Centre for Ophthalmology, University of Tübingen.

## Supplementary Information

Below is the link to the electronic supplementary material.

Supplementary file1 (PDF 566 kb)

## Data Availability

The datasets generated during and/or analysed during the current study are available from the corresponding author on reasonable request.
