# Supplementary material for: Effect of central and peripheral cone- and rod-specific stimulation on the pupillary light reflex
Source: Int Ophthalmol. 2021 Nov 26;42(5):1427–36. doi: 10.1007/s10792-021-02132-1 (PMC9123028; doi:10.1007/s10792-021-02132-1)
Supplement: Supplementary file 1 — Supplementary file1 (PDF 566 kb) [file 10792_2021_2132_MOESM1_ESM.pdf]

SUPPLEMENTARY INFORMATION

Title: Effect of central and peripheral cone- and rod-specific stimulation on the pupillary light reflex

Journal: International Ophthalmology

Anton Sonntag[1], Carina Kelbsch[1,2], Ronja Jung[1,2], Helmut Wilhelm[1,2], Torsten Strasser[1,2,3], Tobias Peters[1], Krunoslav Stingl[1,2,4,*], Barbara Wilhelm[1]

1 Pupil Research Group, Centre for Ophthalmology, University of Tübingen, Tübingen, Germany
2 University Eye Hospital, Centre for Ophthalmology, University of Tübingen, Tübingen, Germany
3 Institute for Ophthalmic Research, Centre for Ophthalmology, University of Tübingen, Tübingen, Germany
4 Centre for Rare Eye Diseases, University of Tübingen, Tübingen, Germany
*Corresponding Author:
Krunoslav Stingl, Pupil Research Group, Centre for Ophthalmology, University of Tübingen, Elfriede-Aulhorn-Str. 7, D-72076 Tübingen, Germany. e-mail: Krunoslav.Stingl@med.uni-tuebingen.de

Supplementary information about Data Management and Statistical Analysis

Before the statistical analysis, artifacts from blinking were removed with an in-house created software via MathWorks, Inc., Matlab, Version R2018b. If no trace was removed because of artefacts, 10 individual traces from each stimulus condition were averaged and the mean pupillary response was used for the following analysis. The mean relative maximal pupillary constriction amplitude (%) (relMCA) was calculated using equation (1):

$$\text{amplitude}_{\text{rel}}(\text{t}) = \frac{\text{baseline}-\text{amplitude}_{\text{absolute}}(\text{t})}{\text{baseline}} * 100 \ (1) \qquad [1]$$

The mean latency to constriction onset (latency) to the different stimuli was determined by calculating the time from start of stimulus until the time of intersection of the baseline and a linear fitting curve through the descending part of the pupillogram using an in-house created algorithm via MathWorks, Inc., Matlab, Version R2018b. Baseline was calculated from 50 points measured between 0 and 500ms. The curve fitted through the descending arm considered 20 individually chosen points around the turning point of the pupillogram. For a visualization of how latency was determined see supplementary Fig. S2.

Analysis and plotting of the relMCAs and latencies was performed with Microsoft Excel 2019. ANOVA, post-hoc test and two-tailed paired t-test were performed with IBM SPSS Statistics Version 25.0.0.1.

Supplementary Figures

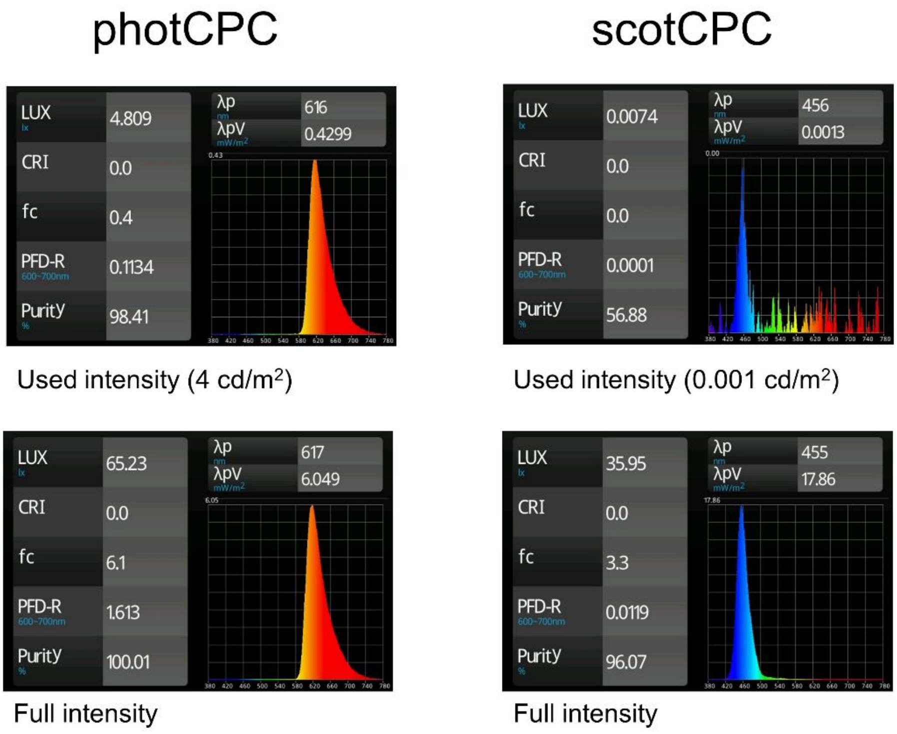

Fig. S1 Energy and spectrum of the applied stimuli at the intensities we used and at full intensity for clarification of the narrowband spectrum. The scattered bars at longer wavelengths at 0.001 cd/m^2 in scotCPC can be interpreted as noise

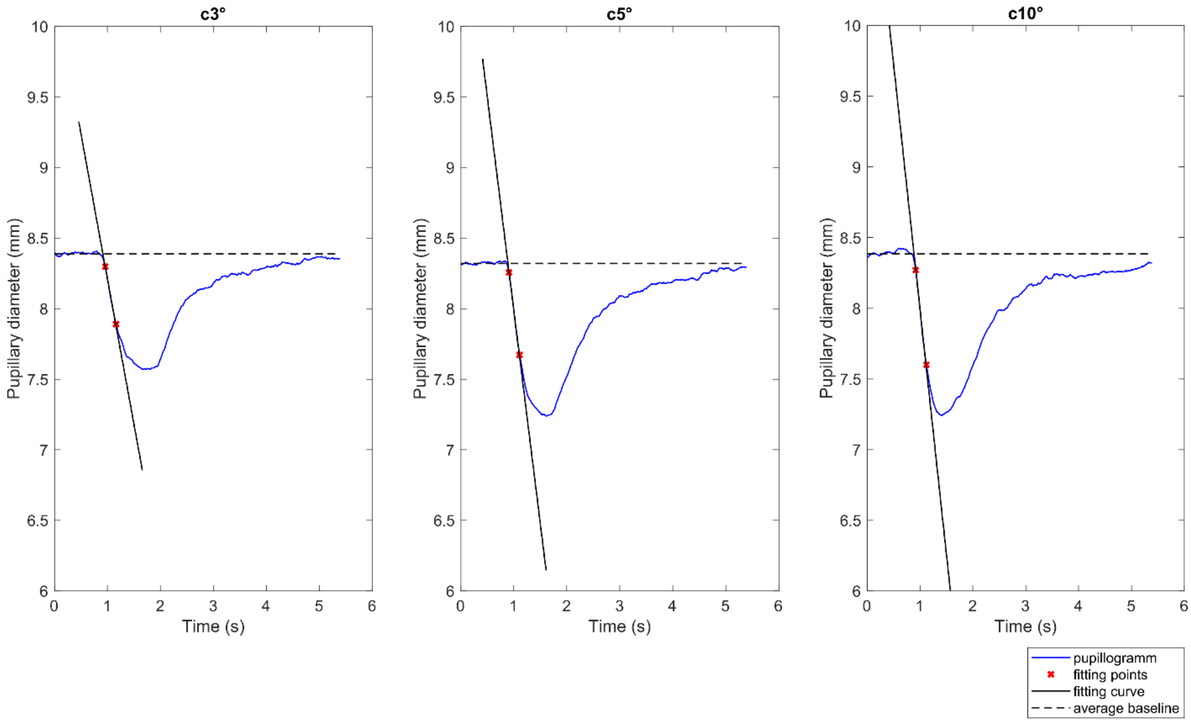

Fig. S2 3 Examples how latency was determined (proband number 10). From left to right there are shown the pupillograms of proband number 10 to the c3°-, c5°- and c10°-stimulus of photCPC (blue line). The dotted black line shows the average pupillary baseline diameter. The solid black line shows the fitting curve through the descending part of the pupillogram. The red crosses show two of the 20 fitting points around the turning point of the pupillogram. Latency is determined as the time from start of stimulus until time of intersection of fitting curve with baseline

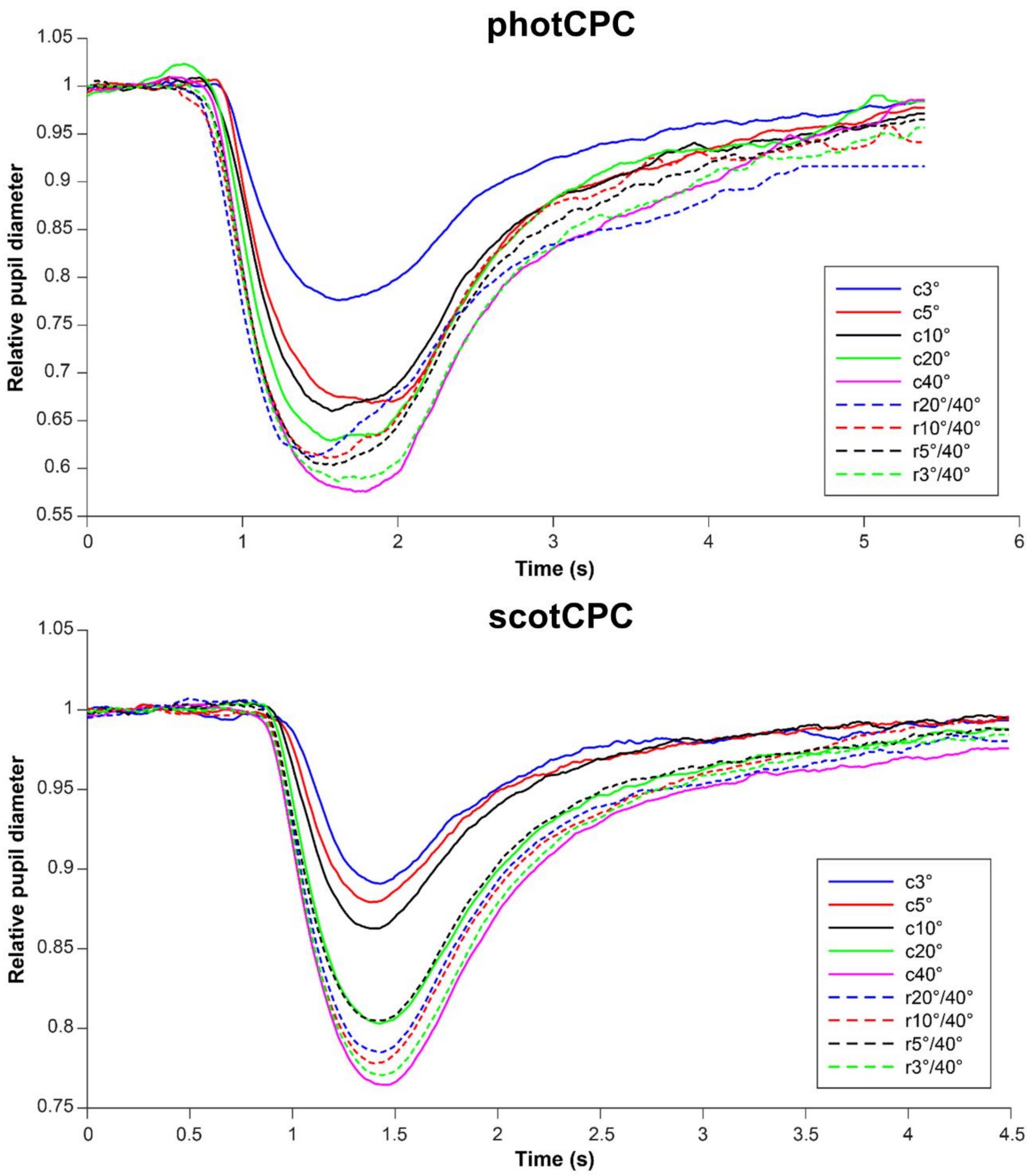

Fig. S3 Exemplary pupil traces of proband number 1 for each stimulus shape for both photCPC and scot CPC. Shapes are explained in Fig. 2 (main text)

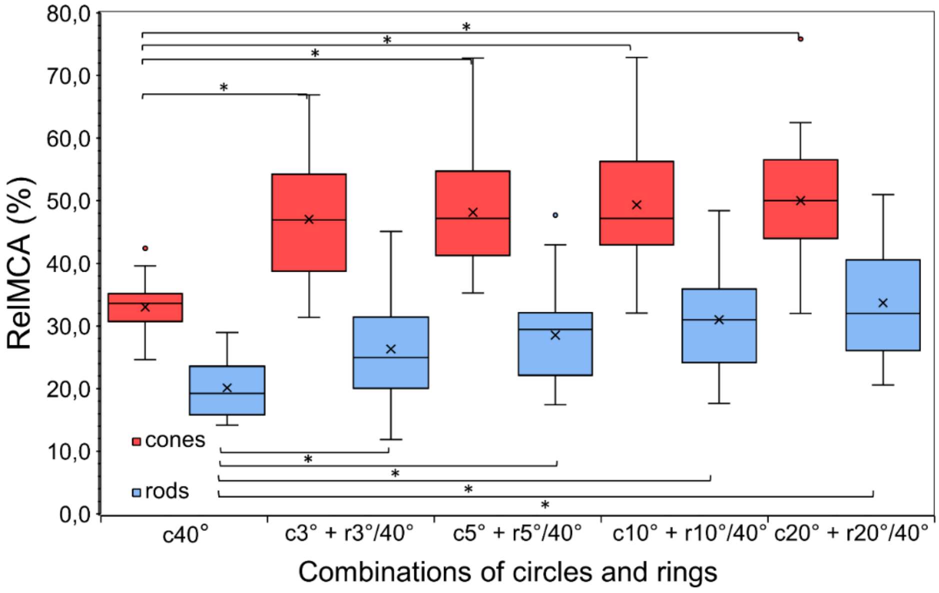

Delta to c40° (abs. % ± SD)	14.0 ± 7.4	15.1 ± 6.9	16.3 ± 6.8	17.0 ± 6.4
	6.2 ± 4.3	8.4 ± 4.2	10.9 ± 4.0	13.6 ± 4.8

Fig. S4 The average (of 10 pupil responses per participant) relMCAs (relative maximal pupillary constriction amplitudes) to the 40° circle stimulus and the summed combinations of stimuli are shown by the box-whisker plots (n = 29). The mean values are shown by the crosses. Responses to cone-specific stimuli are coloured red, responses to rod-specific stimuli are coloured blue. The most important significant differences are shown by the braces. An asterisk means that the difference is statistically significant ($p < 0.001$). The table shows the difference (delta) of the summations to c40°. The analysis of variance (ANOVA) for repeated measurements with a Greenhouse-Geisser correction determined a statistically significant effect of the different combination of circles and rings on the relMCA for cone- and rod-specific stimulation (for both: $p < 0.001$). For both, post-hoc-tests also determined a statistically significant difference between the full-field stimulus and the different summations ($p < 0.001$). Rod-specific responses show a statistically significant difference between the responses to the different summations themselves ($p \leq 0.002$) whereas for cone-specific responses, this difference was not significant.

References

1. Kelbsch C, Stingl K, Kempf M, Strasser T, Jung R, Kuehlewein L, Wilhelm H, Peters T, Wilhelm B, Stingl K (2019) Objective Measurement of Local Rod and Cone Function Using Gaze-Controlled Chromatic Pupil Campimetry in Healthy Subjects. Transl Vis Sci Technol 8 (6):19. https://doi.org/10.1167/tvst.8.6.19
